# The cost of antibiotic resistance depends on evolutionary history in *Escherichia coli*

**DOI:** 10.1186/1471-2148-13-163

**Published:** 2013-08-02

**Authors:** Daniel C Angst, Alex R Hall

**Affiliations:** 1 Institute of Integrative Biology, ETH Zürich, Zürich, CH-8092, Switzerland; 2 Institute of Biogeochemistry and Pollutant Dynamics, ETH Zürich, Zürich, Switzerland; 3 Department of Environmental Microbiology, Eawag, Dübendorf, Switzerland

**Keywords:** Antibiotic resistance, Epistasis, Experimental evolution, *Escherichia coli*

## Abstract

**Background:**

The persistence of antibiotic resistance depends on the fitness effects of resistance elements in the absence of antibiotics. Recent work shows that the fitness effect of a given resistance mutation is influenced by other resistance mutations on the same genome. However, resistant bacteria acquire additional beneficial mutations during evolution in the absence of antibiotics that do not alter resistance directly but may modify the fitness effects of new resistance mutations.

**Results:**

We experimentally evolved rifampicin-resistant and sensitive *Escherichia coli* in a drug-free environment, before measuring the effects of new resistance elements on fitness in antibiotic-free conditions. Streptomycin-resistance mutations had small fitness effects in rifampicin-resistant genotypes that had adapted to antibiotic-free growth medium, compared to the same genotypes without adaptation. We observed a similar effect when resistance was encoded by a different mechanism and carried on a plasmid. Antibiotic-sensitive bacteria that adapted to the same conditions showed the same pattern for some resistance elements but not others.

**Conclusions:**

Epistatic variation of costs of resistance can result from evolution in the absence of antibiotics, as well as the presence of other resistance mutations.

## Background

The persistence of antibiotic-resistant bacteria depends on how resistance, in the form of chromosomal mutations or horizontally acquired elements such as plasmids, affects fitness relative to antibiotic-sensitive genotypes in the absence of antibiotics [1,2]. Recent work shows that the fitness effects of resistance mutations often vary depending on the presence of other resistance mutations on the same genome [3-7]. However, resistance evolution will often be accompanied by the fixation of additional mutations that do not confer resistance but increase fitness in the present environment, either because resistant bacteria evolve in heterogeneous hosts or natural environments where the optimal genotype changes over time, or because the cost of resistance causes selection for compensatory mutations (e.g. [8-13]). Any impact of adaptation in the absence of antibiotics on the subsequent cost of additional resistance mutations therefore potentially modulates costs of resistance in natural populations of pathogens.

**Figure 1:**
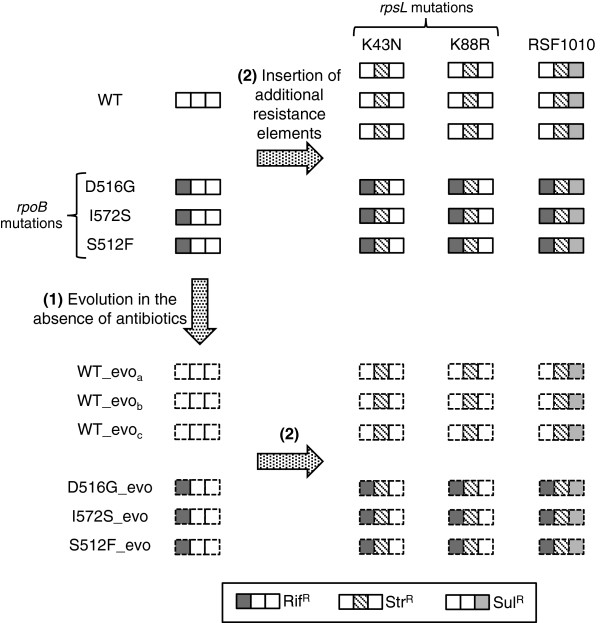
**Construction of different genotypes and associated resistance phenotypes.** (1) The wild type and three Rif^R^ genotypes evolved for approximately 200 generations in liquid LB. (2) Additional resistance elements were inserted in the form of Str^R^ mutations on *rpsL* (K43N or K88R) or a Sul^R^ + Str^R^ plasmid (RSF1010).

Epistatic interactions have been observed among different resistance elements [3-6,14,15], and among different beneficial mutations during adaptation to novel environments [16-19]. However, interactions between the two types of mutations in terms of how they influence fitness in the absence of antibiotics are less clear. In this paper we ask whether adaptation of antibiotic-resistant and sensitive bacteria to the same antibiotic-free environment alters the fitness effects of additional resistance elements that confer resistance against other antibiotics. To test for this possibility, we experimentally evolved rifampicin-resistant (Rif^R^) and rifampicin-sensitive (Rif^S^) *E. coli* in liquid growth medium in the laboratory, before inserting mutations that conferred resistance to streptomycin (Str^R^), shown schematically in Figure 1.

Str^R^ mutations on *rpsL* change the structure of ribosomal protein S12 and interfere with target binding in the presence of streptomycin [20-22]. To determine whether observed effects were specific to *rpsL* mutations, we carried out parallel experiments with a plasmid that confers resistance to sulfonamides and streptomycin (Sul^R^ + Str^R^). The plasmid we used (RSF1010) has a broad host range and encodes resistance to streptomycin by enzymatic modification [23]. Therefore our experiment incorporated multiple distinct antibiotic-resistance mechanisms. We measured the fitness effects of resistance elements by pairwise competition assays, allowing us to quantify the effects of additional resistance elements for Rif^R^ and Rif^S^ bacteria both before and after adaptation. In several cases we found that the cost of a new resistance element was smaller in genotypes that had adapted to our experimental environment compared with the same genotypes without adaptation, although Rif^R^ and Rif^S^ bacteria showed different patterns for some resistance elements.

## Methods

### Bacterial strains and growth conditions

In all experiments we used *E. coli* MG1655 grown at 37°C. To isolate mutants resistant to either rifampicin or streptomycin (Rif^R^ or Str^R^), we plated independent cultures of the wild type onto LB agar supplemented with 50 mg/L rifampicin [24] or 25 mg/L streptomycin. After 24 h incubation at 37°C we picked individual colonies, restreaked to purify genotypes and confirm resistance, before growth for ~2 h in liquid LB and storage at −80°C in 25%v:v glycerol. Rifampicin-resistance mutations were identified by sequencing the central resistance-determining region of *rpoB* as described previously [24]; streptomycin-resistance mutations were identified by sequencing part of *rpsL* using primers *fwd* 5'-ATGATG GCGGGATCGTTG-3' and *rev* 5'-CTTCCAGTTCAGATTTACC-3' [5]*.* All three of the Rif^R^ mutations (D516G, S512F, I572S) and both Str^R^ mutations (K43N and K88R) have been previously associated with resistance to these antibiotics in *E. coli*[5,12,25]. The Sul^R^ + Str^R^ plasmid RSF1010 was obtained from the Deutsche Sammlung von Mikroorganismen und Zellkulturen (DSMZ, Braunschweig, Germany). We constructed double-resistant genotypes, with two resistance mutations or one resistance mutation plus the plasmid, by transduction or transformation as described below.

### Experimental evolution in the absence of antibiotics

We initiated selection lines with each of the three Rif^R^ genotypes (D516G, I572S, S512F) and three replicate lines with the wild type (Figure 1). Each selection line was grown in 100 μl liquid LB medium and diluted 1000-fold into fresh medium approximately every 12 h, as described previously [24]. After 20 transfers, approximately 200 generations, we plated every population onto LB agar and isolated a single colony from each, which we then grew for 2 h in liquid LB and stored at −80°C. We checked for reversion to rifampicin-sensitivity (Rif^S^) in the evolved clones derived from resistant genotypes by plating on LB agar supplemented with rifampicin, but observed none. This confirmed that the colony isolates used in the present study remained Rif^R^, although it does not exclude the possibility of revertants at low frequency in evolved populations. This procedure yielded nine different genotypes in addition to the wild type (left-hand side of Figure 1): three Rif^R^ genotypes that had not adapted to LB, three Rif^R^ genotypes that had adapted to LB (Rif^R^-evolved), and three Rif^S^ genotypes that had adapted to LB (Rif^S^-evolved).

### Addition of streptomycin resistance mutations and plasmid

We used P1 transduction to insert either K43N or K88R into each of the nine genotypes described above, plus three independent replicates of the wild type. We followed [26] with few modifications. Briefly, a lysate of each donor strain was prepared by growth with phage P1 at low multiplicity of infection until lysis was visible, then transduced to the relevant recipient genotype by growth in LB for 20 min, followed by addition of sodium citrate, further incubation for 1 h, plating on LB agar plus streptomycin and sodium citrate, and restreaking three times before isolating a single colony at random and storing at −80°C. All constructed genotypes were verified by resequencing *rpsL*. Transductions were done in a single temporal block; for one genotype (I572S + K88R) a different Str^R^ mutation was acquired during the isolation procedure; for D516G + K43N, no colonies were obtained after repeated attempts; these are excluded from further analysis.

Plasmid RSF1010 is a natural, nontransmissible, broad-host range plasmid of the IncQ incompatibility group conferring resistance to sulfonamide and streptomycin [23]. We extracted the plasmid from cells grown in LB using the PureYield Plasmid Miniprep Kit (Promega). We then transformed the nine isolated genotypes and three replicates of the wild type using TSS Transformation [27]. Briefly, strains were grown in LB to OD_600_ 0.3 and chilled on ice for 10 min before adding an equal volume of ice cold 2× TSS (TSS is LB with 10% w:v PEG8000, 5% v:v DMSO, 50 mM MgSO_4_ at pH 6.5) and incubation on ice for another 30 minutes. We then added 1 ml of competent cells to 1 μl (~100 ng) of the plasmid prep and incubated on ice for one hour. After incubation at 37°C for one hour to allow expression of the resistance genes, we plated cells on LB agar supplemented with 30 mg/L streptomycin. For each transformation a single colony was isolated at random, grown for 2 h in liquid LB and stored at −80°C.

The fitness effects of transduced mutations and the plasmid were reproducible in independently constructed replicates of the wild type: fitness did not differ significantly among different isolates for any of the resistance elements in our experiment (K43N: *F*_2,4_ = 3.00, *P* = 0.16; K88R: *F*_1,4_ = 0.05, *P* = 0.83; plasmid: *F*_2,6_ = 1.33, *P* = 0.33). To check that the plasmid was not lost during competition assays, we plated all competitions that included a plasmid-carrying genotype on tetrazolium arabinose (TA) plates, where streptomycin-resistant genotypes form white colonies as described below, and on LB agar supplemented with 30 mg/L streptomycin, on which plasmid-carrying genotypes can grow but the wild type cannot. We observed approximately the same number of colonies on streptomycin plates as white colonies on TA plates (paired *t*-test: *t*_35_ = 1.49, *P* = 0.14), indicating that the plasmid was maintained throughout the competition.

### Fitness measurements

We measured the competitive fitness of each genotype against a marked strain: *E. coli* K12 MG1655 Δ*ara*, which is otherwise isogenic to the wild type and forms red colonies on TA agar (tryptone 1%, yeast extract 0.1%, NaCl 0.5%, L(+)arabinose 1%, TTC 0.005%). For each competition, we grew independent cultures of both competitors overnight in liquid LB, before mixing them 1:1 v:v and diluting 1000-fold into fresh LB media. We estimated the frequency of each competitor by plating the culture on TA agar before and after two growth cycles using the same protocol as during experimental evolution. We then calculated relative fitness, *w*, as 1 + *s*, where *s* is the selection coefficient *s* = ln(R_final_ / R_initial_) / *t*, where R_final_ and R_initial_ are the ratios of the competing genotypes at the beginning and end of the assay and *t* is assay duration in generations [5,28,29]; in batch culture *t* can be approximated as log_2_(*N*_final_/*N*_initial_), where *N* is total population size. We discounted each score by the cost of the Δ*ara* marker [5], which was not significantly different from zero (−0.025 on average; *P* = 0.06). To calculate the fitness effects of Str^R^ mutations or the plasmid in a given genetic background, we took the difference in fitness (Δ*w*) between the same strain with and without K43N, K88R or the plasmid.

Rif^R^, Rif^R^-evolved and Rif^S^-evolved genotypes lacking either Str^R^ mutations or the plasmid were each assayed in three different blocks: once after the evolution experiment to test for adaptation [24], once alongside genotypes with K43N or K88R, and once alongside genotypes carrying the plasmid. The correlation between fitness scores relative to the wild type measured for these genotypes in different blocks was high (*r*^2^ = 0.91, 0.94, 0.92), and there was no block × genotype interaction (*F*_16,46_ = 1.61, *P* = 0.10), indicating that fitness values of different genotypes relative to each other were repeatable across blocks of assays. In each block, competitions were replicated three times independently, and nine times for the wild type. Additional file 1: Table S1.

### Testing for chaperone (DnaK and GroEL) overproduction

The best-described molecular mechanism for buffering against the fitness effects of deleterious mutations is the overproduction of molecular chaperones [30-32], enzymes that assist in correct protein folding [33]. To determine whether variation of costs of resistance among genotypes in our experiment could be explained by variation in chaperone production, we measured levels of the two key chaperones DnaK and GroEL [34] using western blots. Cells were harvested by centrifugation from mid-exponential cultures, resuspended in 1× SDS-PAGE sample buffer and boiled for 10 minutes. SDS-PAGE and immunoblotting with mouse-monoclonal anti-DnaK and anti-GroEL antibodies (Enzo Life Sciences) was carried out according to standard procedures. ImmunStar Western C Substrate Kit (BioRad) was used for detection in conjuction with horseradish peroxidase-conjugated anti-Mouse lgG secondary antibody (Amersham). Blots were imaged using the ChemiDoc XRS + CCD Sytem (BioRad) and analyzed using ImageJ 1.46 (Rasband, W.S., ImageJ, U. S. National Institutes of Health, Bethesda, Maryland, USA, http://imagej.nih.gov/ij/, 1997–2012.).

### Statistical analyses

We tested for adaptation to LB by a paired *t*-test using the average fitness of each genotype before and after experimental evolution. We tested for variation of the response to selection among genotypes by analysis of variance including genotype and assay block as factors. To test whether adaptation to LB altered the average fitness effects of additional resistance elements (Str^R^ mutations or the plasmid) we conducted paired *t*-tests, taking the average fitness effect (Δ*w*) of each resistance element on each genetic background before and after evolution, measured in the same block of competition assays. We analyzed variation of fitness effects among genotypes that had or had not adapted to LB by analyses of variance with genotype as a factor. Additionally, we tested pairwise epistatic interactions of Rif^R^ mutations with Str^R^ mutations or the plasmid in genotypes that had not adapted to LB. We did this using the multiplicative model described by [5] and results are shown in Additional file 2: Table S2. Finally, to test for differences in levels of chaperones DnaK and GroEL we used pairwise *t*-tests with non-pooled standard deviation, correcting for multiple comparisons using the Bonferroni method.

## Results

### Adaptation in antibiotic-free conditions

**Figure 2:**
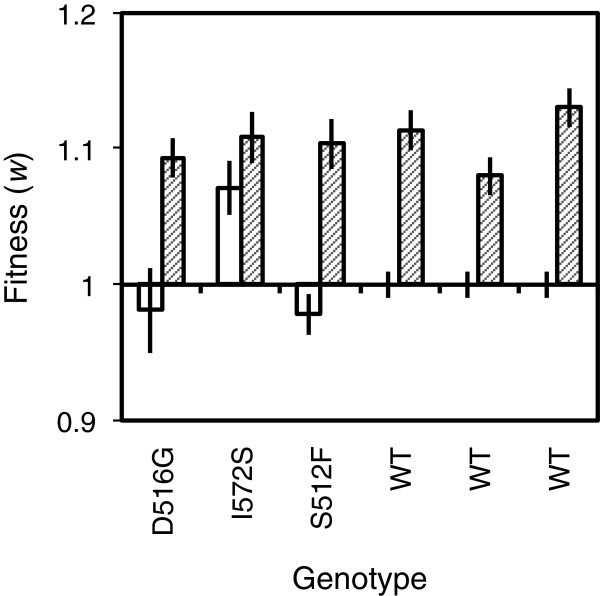
**Adaptation of resistant and sensitive bacteria.** Three Rif^R^ genotypes, denoted on the *x*-axis by their amino acid changes, and three replicates of the wild type (WT) were evolved for approximately 200 generations in antibiotic-free LB medium as described previously [24]. White and hatched bars show scores before and after evolution respectively. Each bar shows average ± s.e. competitive fitness relative to the wild type from nine independent assays, conducted in three temporal blocks; for the wild type before evolution standard error is shown for 27 assays.

Antibiotic-resistant (Rif^R^) bacteria and the antibiotic-sensitive wild type increased in fitness over approximately 200 generations of evolution in LB growth medium (paired *t*-test: *t*_5_ = 6.20, *P* = 0.002; Figure 2). The change in fitness after experimental evolution varied among different evolved genotypes (*F*_5,31_ = 52.99, *P* < 0.0001). Among Rif^R^ genotypes, the response to selection was relatively large for genotypes derived from D516G and S512F, which had lower fitness than the wild type at the start of the experiment, compared with I572S that had a fitness advantage. After experimental evolution, Rif^R^ and Rif^S^ genotypes had similar levels of fitness on average (Welch’s *t*-test: *t*_2.35_ = 0.40, *P* = 0.72; Figure 2). Thus, despite initial variation, resistant and sensitive genotypes adapted to experimental conditions and converged upon similar levels of competitive fitness, as shown previously for a wider range of *rpoB* mutants including the three in this study [24].

### Fitness effects of streptomycin resistance mutations

**Figure 3:**
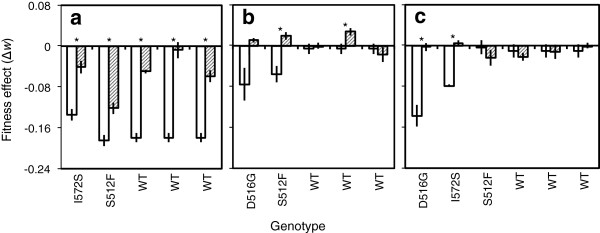
**Effect of adaptation on the cost of additional resistance elements.** Each bar shows the fitness effect of **(a)***rpsL* K43N, **(b)***rpsL* K88R or **(c)** plasmid RSF1010 either before (white bars) or after (hatched bars) adaptation to LB growth medium. Asterisks denote statistically significant effects of adaptation, as determined by pairwise *t*-tests assuming unequal variances. Each bar shows mean ± s.e. for three independent assays; for the wild type before evolution, bars show the average of three independently constructed genotypes, each assayed three times.

The fitness effects of Str^R^ mutations were smaller on average in genotypes that had adapted to LB compared to the same genotypes without adaptation (paired *t*-test: *t*_8_ = 4.83, *P* = 0.001; Figure 3a,b). Across all genetic backgrounds, K43N was more costly on average than K88R (mean fitness effect ± s.d.-K43N:-0.11 ±0.07; K88R:-0.01 ±0.04; Figure 3a,b).

For K43N, all of the Rif^R^-evolved and Rif^S^-evolved genotypes we tested paid a smaller cost of resistance than the same genotypes without evolution in LB (Figure 3a). Among genotypes that had not adapted to LB, the cost was similar in S512F and the wild type, but comparatively small for I572S. This represents antagonistic pairwise epistasis between I572S and K43N: the cost of having both mutations was smaller than expected from their independent effects on the wild type (Additional file 2: Table S2). Among evolved genotypes, the cost of K43N also varied depending on genetic background (*F*_4,10_ = 12.84, *P* < 0.001), but in every case it was less costly than without evolution in LB.

When streptomycin resistance was due to K88R, the fitness cost was smaller after adaptation to LB for both of the Rif^R^ genotypes we tested, although this only represented statistically significant variation for S512F (Figure 3b). The fitness effect of K88R varied among genotypes that had not adapted to LB (*F*_2,9_ = 4.67, *P* = 0.04), being costly on average in Rif^R^ genotypes but not the wild type. K88R was also approximately neutral for two out of three evolved wild type (Rif^S^) genotypes, and for the remaining one it had a small positive fitness effect. Consequently, adaptation to LB had no effect on average for the cost of K88R in Rif^S^ genotypes (*F*_1,3_ = 0.31, *P* = 0.62). By contrast, K88R was costly to Rif^R^ genotypes before, but not after experimental evolution in LB.

In summary, for one Str^R^ mutation (K43N) the fitness cost was consistently lower for evolved genotypes than the same genotypes without adaptation to LB. A similar effect was observed for the other Str^R^ mutation on Rif^R^ genetic backgrounds, but not for Rif^S^ genotypes, where K88R was approximately neutral both before and after evolution in LB on average. Thus, the fitness effects of Str^R^ mutations varied epistatically depending on both adaptation in the absence of antibiotics and on the presence of other resistance mutations.

### Fitness effects of an antibiotic-resistance plasmid

Insertion of the plasmid RSF1010, conferring resistance to sulfonamides and streptomycin, had a marginal cost on average (mean fitness effect ± s.d. = −0.03 ± 0.05), but this varied considerably among genotypes depending on adaptation to LB and Rif^R^ mutation (Figure 3c). There was no difference on average between the cost of RSF1010 in genotypes that had evolved in LB and those that had not (paired *t*-test: *t*_5_ = 1.29, *P* = 0.25), although in some cases the fitness effect of the plasmid was clearly lower in evolved genotypes. Specifically, RSF1010 was less costly for genotypes with Rif^R^ mutations D516G or I572S after they had evolved in LB (Figure 3c), but there was no difference for S512F or any of the Rif^S^ evolved genotypes. This variation was driven by a comparatively large fitness cost in two of the unevolved Rif^R^ genotypes, reflecting negative pairwise epistasis between the plasmid and D516G and I572S (Additional file 2: Table S2). Thus, the plasmid was costly to D516G and I572S before but not after evolution in antibiotic-free LB; other genotypes paid a comparatively small cost of carrying the plasmid.

### Buffering of fitness effects is not due to overexpression of chaperones

The phenotypic effects of deleterious mutations can be buffered by overproduction of molecular chaperones, of which GroEL and DnaK are the two most important and best studied [32,34]. However, we found no upregulation of either chaperone in evolved genotypes (pairwise *t*-tests: all *P* values > 0.075; Additional file 3: Figure S1).

## Discussion

We measured the fitness effects of three different antibiotic-resistance elements (two Str^R^ mutations and a Sul^R^ + Str^R^ plasmid) in Rif^R^ and Rif^S^ genotypes that had or had not adapted to antibiotic-free growth medium. In cases where additional resistance elements were costly to genotypes that had not evolved in LB, the cost was consistently smaller after adaptation to our experimental environment. For one of the Str^R^ mutations this pattern was consistent across Rif^R^ and Rif^S^ genotypes, suggesting that the relatively small cost of new resistance mutations after adaptation in the absence of antibiotics is not specific to resistant bacteria. For the other Str^R^ mutation and the plasmid, the cost was greatest in Rif^R^ genotypes that had not adapted to LB, and evolved genotypes showed a small cost or even a benefit of resistance by comparison. These results show that evolution in the absence of antibiotics can alter the fitness effects of new resistance elements, but this pattern depends on the identity of the new mutation and on the presence of other resistance mutations.

Consistent with previous studies [3,5,6,15,35], we found pairwise epistatic interactions among resistance elements on the same genetic background: out of seven multiple-resistant genotypes with a Rif^R^ mutation and either an Str^R^ mutation or the plasmid, three showed significant deviations from a multiplicative model of fitness (Additional file 2: Table S2). Moreover, our finding that evolution in the absence of antibiotics alters the fitness effects of additional resistance elements suggests that epistatic variation of costs of resistance can also be caused by beneficial mutations that fix in the absence of antibiotics.

To our knowledge, the best characterized mechanism that actively buffers against deleterious mutations is over-expression of molecular chaperones [34,36,37]. This did not explain our findings: the two main chaperones, DnaK and GroEL, were not overproduced in evolved genotypes. This is perhaps unsurprising, given that selection for chaperone-mediated buffering is expected to depend on intense genetic drift [38] or high mutation rates [39,40], which is supported by experiments with viruses [41]. In the absence of direct selection for a buffering mechanism, the impact of adaptation on the cost of Str^R^ mutations and the plasmid is probably an indirect effect of mutations that were under positive selection for their effects on growth in LB. By analogy, *rpoB* mutations that fix under selection for rifampicin resistance have wide-ranging effects on the bacterial phenotype, owing to direct effects on the function of RNA polymerase and pleiotropic effects on the expression of other genes [42-44]. This is associated with altered growth phenotypes in unselected environments [44,45] and epistatic interactions with other resistance mutations [5,6]. Although we lack a physiological understanding of adaptation to LB, it is known to alter growth phenotypes in unselected environments to a similar degree as *rpoB* mutations in this experimental system [24]. This is consistent with beneficial mutations fixed in LB having indirect phenotypic effects that are unrelated to improved growth in LB but may generate epistatic interactions.

Our experiments included a limited set of resistance elements (three different types, including chromosomal mutations and a plasmid). Therefore we do not draw any general conclusions about how adaptation will influence the cost of new resistance elements. However, our results do show that adaptation in the absence of antibiotics can cause epistatic variation of fitness costs. In support, a recent study demonstrated variation of the fitness effects of rifampicin-resistance mutations among antibiotic-sensitive *E. coli* genotypes [46]. This is consistent with epistatic variation of fitness costs due to mutations at loci unrelated to drug resistance. We also stress that with only one selection line for each Rif^R^ genotype, variation in the outcomes of evolution among evolved Rif^R^ genotypes does not necessarily reflect an effect of starting genotype. Stochastic processes during adaptation, including the random appearance and loss of beneficial mutations [47], will also contribute to variation among individual selection lines. Therefore, while we found differences on average between Rif^R^ and Rif^S^ evolved genotypes, and between evolved and nonevolved genotypes, we did not test whether the effect of adaptation varies among genotypes with different rifampicin-resistance mutations.

Directions for further work include identifying the physiological mechanisms by which adaptation to antibiotic-free conditions can alter the costs of new resistance elements, and how general such effects are. For example, expression profiling of resistant and sensitive genotypes that have and have not been experimentally evolved could be used to find cellular functions that are altered by resistance mutations and adaptation, both independently and in combination. To determine whether adaptation has a general effect on the cost of additional resistance mutations would require experiments with other model organisms, antibiotics and growth media. In particular, testing whether compensatory mutations that ameliorate the deleterious effects of one resistance mutation [8,10-13] can buffer against the effects of resistance elements at other loci would be relevant to the broader question of how selection acts on antibiotic resistance across genetic backgrounds.

## Conclusions

We observed epistatic variation of the fitness costs associated with antibiotic resistance, stemming from interactions between different resistance elements as observed previously [3,5,6] and from interactions between resistance elements and beneficial mutations that fixed during adaptation to drug-free conditions. This is potentially relevant for the cost of resistance in real populations of pathogenic bacteria. In chronic infections, such as those caused by *Pseudomonas aeruginosa* in cystic fibrosis patients, bacteria fix multiple mutations during a single infection, many of which are unrelated to resistance evolution [48-50]. Other pathogens such as *Mycobacterium tuberculosis*, which often carry antibiotic resistance elements, also fix mutations that are beneficial in the absence of antibiotics [51]. If such mutations influence the cost of subsequent resistance mutations, as they did in our experiment, then a better understanding of epistasis between resistance elements and other types of mutations will be important for predicting the likelihood that multi-drug resistant bacteria will persist following different types of treatment.

## Competing interests

The authors declare that they have no competing interests.

## Authors’ contributions

DCA and ARH designed and performed the experiments, analyzed data and wrote the paper. Both authors read and approved the final manuscript.

## Supplementary Material

Additional file 1: Table S1 Relative Fitness of all Genotypes. Click here for file

Additional file 2: Table S2 Epistasis between RifR mutations, StrR mutations and plasmid RSF1010. Click here for file

Additional file 3: Figure S1 Chaperone levels in different genotypes. Click here for file
