# Supplementary material for: The cost of antibiotic resistance depends on evolutionary history in Escherichia coli
Source: BMC Evol Biol. 2013 Aug 2;13:163. doi: 10.1186/1471-2148-13-163 (PMC3751127; doi:10.1186/1471-2148-13-163)
Supplement: Additional file 1: Table S1 — Relative Fitness of all Genotypes. [file 1471-2148-13-163-S1.pdf]

TABLE S1. Relative fitness of all genotypes.

Fitness was measured by competition assays against the wild type. Rifampicin-resistance (Rif^R) mutations are given by their amino acid changes on *rpoB*; additional resistance elements (Str^R or Sul^R+Str^R) are given either as amino acid changes on *rpsL* (K43N or K88R) or RSF1010 for the plasmid. Independently generated isolates of the wild type (evolved in LB or with additional resistance elements) are denoted *a-c* in *Genotype name*. Scores are mean ±s.e. from three independent replicates, or nine replicates across three blocks for genotypes with no additional resistance elements, and 27 replicates across three blocks of assays for the wild type. For WTc+K43N data are from one competition assay.

Genotype name	Rif^R mutation	Evolved in LB	$Additional^R$ element	Fitness	s.e.
D516G+K88R	D516G	no	K88R	0.90	0.03
D516G	D516G	no	none	0.98	0.03
D516G+plasmid	D516G	no	RSF1010	0.91	0.02
D516G_evo+K88R	D516G	yes	K88R	1.06	0.00
D516G_evo	D516G	yes	none	1.09	0.01
D516G_evo+plasmid	D516G	yes	RSF1010	1.15	0.01
I572S+K43N	I572S	no	K43N	0.94	0.01
I572S	I572S	no	none	1.07	0.02
I572S+plasmid	I572S	no	RSF1010	1.06	0.00
I572S_evo+K43N	I572S	yes	K43N	1.02	0.01
I572S_evo+K88R	I572S	yes	K88R	1.09	0.01
I572S_evo	I572S	yes	none	1.11	0.02
I572S_evo+plasmid	I572S	yes	RSF1010	1.18	0.01
S512F+K43N	S512F	no	K43N	0.81	0.01
S512F+K88R	S512F	no	K88R	0.93	0.02
S512F	S512F	no	none	0.98	0.02
S512F+plasmid	S512F	no	RSF1010	1.00	0.01
S512F_evo+K43N	S512F	yes	K43N	0.95	0.01
S512F_evo+K88R	S512F	yes	K88R	1.09	0.01
S512F_evo	S512F	yes	none	1.10	0.02
S512F_evo+plasmid	S512F	yes	RSF1010	1.15	0.02
WTa+K43N	none	no	K43N	0.82	0.01

WTb+K43N	none	no	K43N	0.81	0.01
WTc+K43N	none	no	K43N	0.86	
WTa+K88R	none	no	K88R	1.00	0.01
WTb+K88R	none	no	K88R	0.99	0.02
WT	none	no	none	1.00	0.01
WTa+plasmid	none	no	RSF1010	0.98	0.00
WTb+plasmid	none	no	RSF1010	0.97	0.01
WTc+plasmid	none	no	RSF1010	1.02	0.03
WTa_evo+K43N	none	yes	K43N	1.04	0.00
WTb_evo+K43N	none	yes	K43N	1.03	0.02
WTc_evo+K43N	none	yes	K43N	1.04	0.01
WTa_evo+K88R	none	yes	K88R	1.09	0.01
WTb_evo+K88R	none	yes	K88R	1.07	0.01
WTc_evo+K88R	none	yes	K88R	1.08	0.01
WTa_evo	none	yes	none	1.11	0.01
WTb_evo	none	yes	none	1.08	0.01
WTc_evo	none	yes	none	1.13	0.01
WTa_evo+plasmid	none	yes	RSF1010	1.16	0.01
WTb_evo+plasmid	none	yes	RSF1010	1.12	0.01
WTc_evo+plasmid	none	yes	RSF1010	1.18	0.01
