# Supplementary material for: The cost of antibiotic resistance depends on evolutionary history in Escherichia coli
Source: BMC Evol Biol. 2013 Aug 2;13:163. doi: 10.1186/1471-2148-13-163 (PMC3751127; doi:10.1186/1471-2148-13-163)
Supplement: Additional file 2: Table S2 — Epistasis between RifR mutations, StrR mutations and plasmid RSF1010. [file 1471-2148-13-163-S2.pdf]

TABLE S2. Epistasis between Rif^R mutations, Str^R mutations and plasmid RSF1010.

We used a multiplicative model to estimate epistasis, ε, for each pair of mutations or each mutation+plasmid combination in genotypes that had not adapted to LB; $\varepsilon = W_{AB}W_{ab} - W_{Ab}W_{aB}$, where W_{ij} is the fitness of genotypes with alleles *i* and *j* and capitals denote wild-type alleles[1]. To test whether epistasis values were significantly different from zero we used the error propagation method described in [1]; asterisks denote significant deviations from multiplicative fitness effects. To estimate the fitness effect of a given resistance element in the wild type, we pooled the data for the three independently constructed strains, because they showed no significant variation in fitness and we therefore assume them to be genetically identical. To test whether these results were robust to a different model of epistasis, we estimated ε for the same data using an additive model, where $\varepsilon = \alpha_{ab} - (\alpha_{Ab} + \alpha_{aB})$, where α is the fitness effect of having alleles *i* and *j* [1, 2]. Under an additive model we obtained the same qualitative results as with the multiplicative model, and scores from different models were closely correlated ($r^2 = 0.99$).

1. Trindade S, Sousa A, Xavier KB, Dionisio F, Ferreira MG, Gordo I: **Positive epistasis drives the acquisition of multidrug resistance**. *PLoS Genetics* 2009, **5**(7):e1000578.
2. Phillips PC: **Epistasis - the essential role of gene interactions in the structure and evolution of genetic systems**. *Nature Reviews Genetics* 2008, **9**(11):855-867.

Double mutant	Observed fitness (w)	s.d. w	Epistasis (ε)	s.d. ε
D516G+K88R	0.897	0.055	-0.069	0.082
I572S+K43N	0.936	0.019	0.057*	0.048
S512F+K43N	0.806	0.019	-0.007	0.053
S512F+K88R	0.935	0.028	-0.050	0.064
D516G+plasmid	0.909	0.037	-0.126*	0.115
S512F+plasmid	1.000	0.024	0.007	0.063
I572S+plasmid	1.056	0.004	-0.067*	0.065
