# Supplementary material for: The cost of antibiotic resistance depends on evolutionary history in Escherichia coli
Source: BMC Evol Biol. 2013 Aug 2;13:163. doi: 10.1186/1471-2148-13-163 (PMC3751127; doi:10.1186/1471-2148-13-163)

Figure S1. **Chaperone levels in different genotypes.**

Protein levels were determined by western blots for all genotypes relative to the wild type (1.0). Data are shown for two molecular chaperones (a) DnaK and (b) GroEL for each genotype (*x*-axis) before (white bars) and after (hatched bars) evolution in LB. Bars show standard errors. Neither chaperone was overexpressed after evolution (main text).

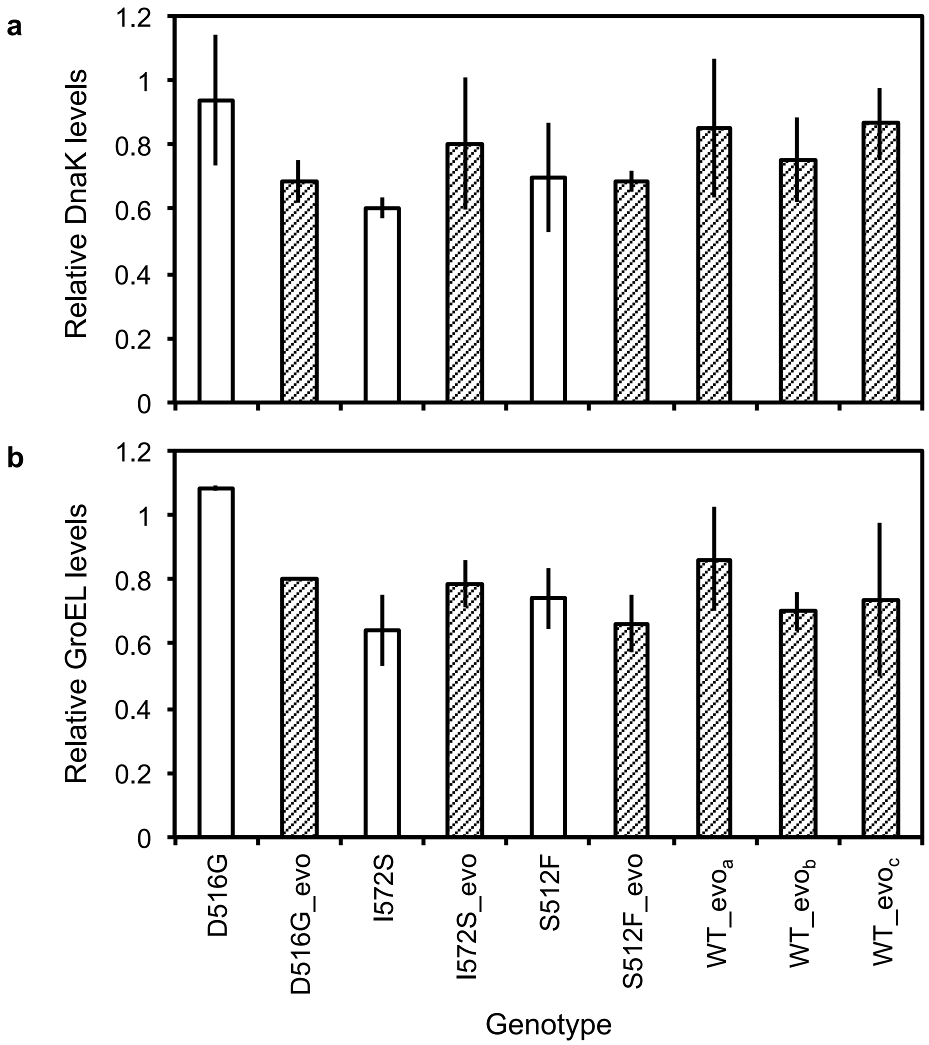

Supplement: Additional file 3: Figure S1 — Chaperone levels in different genotypes. [file 1471-2148-13-163-S3.pdf]
